# Prevalence and Factors Associated with Gaming Disorder in Latin America and the Caribbean: A Systematic Review

**DOI:** 10.3390/ijerph191610036

**Published:** 2022-08-15

**Authors:** Akram Hernández-Vásquez, Rodrigo Vargas-Fernández, Fabriccio J. Visconti-Lopez, Daniel Comandé, Guido Bendezu-Quispe

**Affiliations:** 1 Centro de Excelencia en Investigaciones Económicas y Sociales en Salud, Vicerrectorado de Investigación, Universidad San Ignacio de Loyola, Lima 15024, Peru; 2 Facultad de Ciencias de la Salud, Universidad Científica del Sur, Lima 15067, Peru; 3 Facultad de Ciencias de la Salud, Universidad Peruana de Ciencias Aplicadas, Lima 15067, Peru; 4 Instituto de Efectividad Clínica y Sanitaria (IECS-CONICET), Buenos Aires C1414CPV, Argentina; 5 Centro de Investigación Epidemiológica en Salud Global, Universidad Privada Norbert Wiener, Lima 15046, Peru

**Keywords:** gaming disorder, internet addiction disorder, systematic review, Latin America

## Abstract

We aimed to determine the prevalence and factors associated with gaming disorder (GD) in the population of Latin America and the Caribbean (LAC). A systematic review was performed (PROSPERO protocol registration: CRD42021230565). We included studies that identified participants with GD and/or factors associated with this condition, reported the prevalence of GD, or contained data that assisted in its estimation, were published after 2013 (the year of inclusion of GD in the Fifth Edition of the Diagnostic and Statistical Manual of Mental Disorders) and were carried out in a population residing in an LAC country. Evaluation of the quality of the studies was carried out using the Joanna Briggs Institute Critical appraisal checklist tool. A qualitative synthesis of the data was performed. Of the total of 1567 records identified, 25 passed the full-text review phase, and 6 met the selection criteria. These studies were published between 2018 and 2021 and had a cross-sectional design (three in Brazil, one in Ecuador, Mexico, and the other was multi-country, including a LAC country [Peru]). The prevalence of GD ranged from 1.1% to 38.2%. The three studies in Brazil had the highest figures of GD prevalence (20.4–38.2%). Four studies evaluated factors associated with GD. Characteristics regarding the game (type), pattern of use (hours played), as well as gender (higher in men), tobacco and alcohol consumption, poor interpersonal relationships, and the presence of mental disorders were found to be associated with GD in LAC. Evidence on the prevalence and factors associated with GD in LAC is limited. Studies on GD in LAC evaluate different population subgroups, describing a wide prevalence of this condition (present in up to 38 out of 100 evaluated). Characteristics such as the type and hours of use of the games, sociodemographic data, lifestyles, interpersonal relationships, and the presence of mental disorders increase the probability of presenting GD.

## 1. Introduction

Gaming disorder (GD) is an addictive behavior that involves impaired control over gaming, increased priority given to gaming, persistent gaming behavior despite negative psychosocial consequences, and impaired functioning, social interactions, and educational and work activities for at least 12 months [1,2]. GD was first included in the Fifth Edition of the Diagnostic and Statistical Manual of Mental Disorders (DSM-5) in 2013 and was recently classified as a disease in The International Classification of Diseases 11th Revision (ICD-11) [3]. Although GD affects approximately 3% of the world’s population [4], the prevalence of GD varies among countries (ranging between 1.2 and 1.6% in Europe [5,6], between 0.3 and 1.0% in the United States [7], and between 1.6 and 18.4% in Asia) [8,9], and men have a 2.5-fold greater risk of presenting GD compared to women [4]. These differences can be attributed to the methodological and population characteristics included in the epidemiological studies and the cultural and demographic factors that differ among the populations [10,11,12].

According to the biomedical literature, there are factors associated with GD that are related to the demographic characteristics of the person (male sex, younger age, people with marital difficulties, and lack of family harmony) and their environment [13]. Environment-related factors are linked to interpersonal relationships and educational or social activities (problems with peers, greater number of friends, lower educational and professional performance, and lack of social skills, competence, and integration) and to the characteristics of the personality and mental and physical health conditions (impulsivity, extroversion, aggressiveness, violence, low self-esteem, low personal satisfaction, attention deficit disorder, depression, anxiety, sleep problems, and addiction to psychoactive substances) [13]. The persistence of these factors depends on the sociodemographic and cultural context of the population studied, and even more so when there are other factors such as poverty and violence (especially in low- and middle-income countries such as Latin America and the Caribbean [LAC]) that increase the risk of suffering from mental health disorders [14].

In LAC, approximately 650 million people are distributed unequally in 33 countries that make up this region, where Brazil and Mexico have the largest number of inhabitants [15]. In this region, the majority of the people speak Spanish, and they share characteristics such as a high rate of unemployment, poverty, exposure to violence, limited human and economic resources in health systems, and a high prevalence and burden of disease due to mental health disorders [16,17]. In LAC, by 2020, there were 272.5 million players, being one of the regions with the highest growth in the number of gamers and spending in games worldwide [18]. In this region, the prevalence of GD varies between countries. Ecuador has a prevalence of 1.13% [19], Mexico of 5.2% [20], and in Brazil, the prevalence of GD ranges between 20.4% and 38.2% [21,22,23]. These prevalence figures in LAC countries may be due to the limitations of the studies (because they were carried out in different contexts, using different instruments or measurements for the diagnosis of GD) and limitations that these regions present, such as low investment in prevention programs for mental health problems, insufficient human resources, low coverage of mental health care, and poor health systems [11,24,25]. In addition, in LAC, there is a problem in decision making and the impact of mental health policies due to the scarcity of clinical-epidemiological information on mental health disorders [26]. These limitations, together with the sociodemographic and cultural differences between and within countries [11], generate variations in the figures of the prevalence of GD in this region and increase the risk of presenting this disorder.

In general, most epidemiological studies that explore the prevalence and factors associated with GD were carried out in regions of Europe and Asia [4]. In LAC, the studies performed focused on the prevalence and associated factors in specific populations without considering the epidemiological characteristics and the sociodemographic, economic, and social determinants presented by the diverse populations that make up LAC [11], and particular characteristics concerning the use of video games in the region (in LAC, there is a growth in the use of online games due to an increase in internet coverage in the region, which makes it easier to participate in these recreational activities in countries whose laws of importation make the purchase of imported video game equipment more expensive compared to other regions) [18]. This is especially important given that this region presents limitations in access, coverage, and execution of prevention and treatment programs for mental health disorders. Moreover, these limitations were accentuated by the coronavirus disease (COVID-19) pandemic, which generated a greater risk and disease burden due to mental health problems. Therefore, this study aimed to summarize the evidence available on the prevalence and factors associated with GD in the LAC population.

## 2. Materials and Methods

This systematic review was reported according to the guidelines of the Meta-analysis of Observational Studies in Epidemiology (MOOSE) and guidelines for Preferred Reporting Items for Systematic Review and Meta-Analysis (PRISMA 2020) [27,28]. The protocol was prospectively registered in the Prospective International Registry of Systematic Reviews (PROSPERO) database (registration number CRD42021230565).

### 2.1. Data Sources and Search Strategy

A condition, context, and population (CoCoPop) process was developed for this systematic review [29] which was as follows: the condition was people with GD and/or associated factors; the context was any setting of LAC countries; and the population was any age, gender or ethnic group. We searched for all potentially relevant studies published from the date of the creation of the bibliographic databases used until 30 December 2021. For this, the largest academic databases worldwide (PubMed, Scopus, Embase, Global Health, CINAHL) and a specialized database in psychology (PsycINFO) were used. Likewise, the LILACS database was used, which indexes the largest number of peer-reviewed journals from Latin American and Caribbean countries and includes some regional databases [30]. The search strategy was carried out by an experienced medical research librarian (DC) and validated by the research team (see strategies in Supplementary Table S1). We did not apply design, language, or time restrictions. In addition to the electronic search, we reviewed the reference lists of all the studies included potentially eligible publications.

The records found in the electronic search were imported to the EndNote X9 (Philadelphia, PA, USA) reference management software, and all duplicate records were removed following the procedures described by Bramer et al. [31].

### 2.2. Identification and Selection of Studies

Peer-reviewed studies were included if they met the following criteria: (a) evaluated participants for any GD based on any (i.e., DSM/ICD) criteria and/or their associated factors; (b) reported the prevalence of GD or contained data that assists in its estimation; (c) were published from 2013, which corresponds to the year in which the GD was first included in the DSM-5, (d) carried out in a population residing in countries of LAC. In addition, we excluded articles that: (a) did not report GD diagnostic criteria; (b) were not published in English, Spanish, or Portuguese; (c) included review articles, reports, book chapters, editorials, comments, conference abstracts, or letters. If there were two or more publications with the same population, the publication with the largest sample size or information about the prevalence and/or factors associated with GD was included.

Two review authors (FJVL and GBQ) independently assessed the titles and abstracts of all the registries identified in the search and met the inclusion criteria using the Rayyan web application [32]. Discrepancies in decisions were resolved by consensus or discussion with a third author (AHV). All the registries included proceeded to the full-text evaluation phase by two authors (FJVL and GBQ). Disagreements about the inclusion or exclusion of the article were resolved with a third author (AHV).

### 2.3. Outcome Measures

The main outcomes of this review were as follows: (a) prevalence of GD in the population residing in LAC countries, and (b) the factors associated with GD.

### 2.4. Data Extraction

Data extraction was carried out by two authors independently (FJVL and GBQ) using a data extraction form in Excel 2020, Microsoft 365 (Washington, DC, USA), and any discrepancies were resolved by consensus or discussion with a third author (AHV). Data were extracted, including the first author, year of publication, country of study, the language of publication, study design, setting, type of participants, the prevalence of GD, factors associated with GD, and the methods used to diagnose GD, including diagnostic methods, techniques for measurement, and threshold values, sample size, population characteristics, and funding/conflict of interest.

### 2.5. Evaluation of the Quality of the Studies

The JBI Critical appraisal checklist (https://jbi.global/critical-appraisal-tools (accessed on 25 January 2022)) was used to assess the quality of the studies included [33]. This assessment was conducted independently by two authors (FJVL and GBQ), and a consensus was reached between the two authors in case of disagreement.

### 2.6. Data Synthesis

We utilized a narrative synthesis approach. Descriptive tables were constructed with information on the prevalence and associated factors from studies conducted in LAC. Descriptive summaries of the results for the population of LAC, including country, sex, age, measurement instrument, and associated factors, were reported.

### 2.7. Ethical Considerations

The study did not require the approval of an ethics committee because it was an analysis of aggregated secondary data in the public domain and did not identify the evaluated participants.

## 3. Results

**Figure 1:**
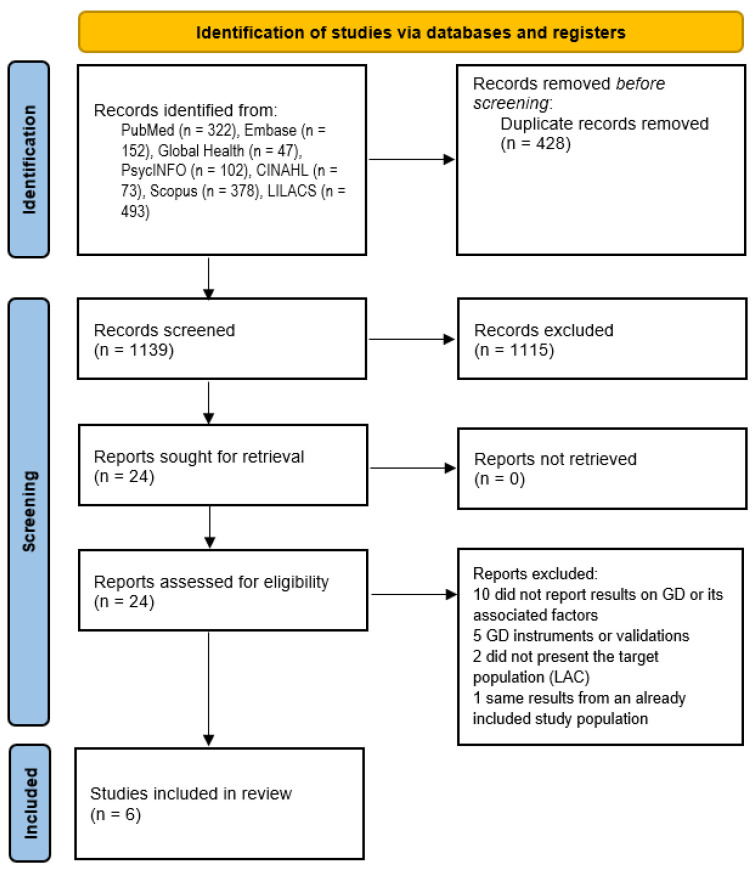
PRISMA 2020 Flow diagram of study selection. GD: gaming disorder, LAC Latin America and The Caribbean.

Of a total of 1567 potential records, 428 duplicate records were removed. In total, 1139 articles were evaluated by reviewing the title and abstract, and 24 passed to the full-text review phase [19,20,21,22,23,34,35,36,37,38,39,40,41,42,43,44,45,46,47,48,49,50,51,52]. Finally, six met the selection criteria and were included in the systematic review. Regarding the excluded articles, ten were not included because they did not report results on GD [34,35,36,37,38,39,40,41,42,52], five studies were excluded because they were instruments or validations of GD [23,43,44,45,46], two studies did not include the target population (LAC) [47,48], and one study was excluded because it had the same results as an already included study population [49] (Figure 1).

### 3.1. Characteristics of Studies Included

Table 1 summarizes the characteristics of the studies included. Data on the prevalence of GD were obtained from six studies involving 15,713 people in four countries, published between 2018 and 2021. All studies had a cross-sectional design. Three studies were conducted in Brazil [21,22,23], one in Ecuador [19], one study was performed in Mexico [20], and there was one multi-country study in which one of the countries included was Peru (for this study, only information regarding Peruvian participants in the multi-country study was used) [47]. Regarding the language of the studies, five studies were published in English [20,21,22,23,47], and one study was in Spanish [19].

Among the demographic characteristics of the studies included, five studies reported the age of the participants [19,21,22,23,47]; however, the reporting of age was heterogeneous across studies. Furthermore, all studies included a predominantly male population. Regarding the context in which the studies were carried out, three studies were performed in schools [19,21,22], one study at a university [20], one study in colleges and universities [23], and one study was conducted on virtual platforms [47].

### 3.2. Instruments Used for the Identification of Gaming Disorder Cases

Regarding the identification of GD cases, all the studies used previously validated instruments to diagnose this disorder. One study used the Diagnostic and Statistical Manual of Mental Disorders, 5th Edition-Internet Gaming Disorder (DSM-5 IGD) scale, while another study used a self-report instrument based on the DSM-5 IGD criteria [20,22], one study used the Internet Gaming Disorder Test (IGD-20) [19], one study used the Gaming Addiction Scale (GAS) [21], one study used the Ten-Item Internet Gaming Disorder Test (IGDT-10) [47], and one study used the Brazilian version of the Internet Gaming Disorder Scale-Short-Form (IGDS9-SF) [23] (Table 2).

### 3.3. Results on the Prevalence of Gaming Disorder

All the studies included estimated the prevalence of GD. In this regard, the prevalence of GD ranged between 1.1 and 38.2%, with the study by Andrade et al. in an Ecuadorian school population having the lowest prevalence [19]. In contrast, the study conducted by Severo et al. with a population of schoolchildren and university students from the South of Brazil reported the highest prevalence [23]. Three studies conducted in Brazil had the highest prevalence of GD in the region, ranging between 20.4 and 38.2% [21,22,23]. On the other hand, a study by Borges et al. on university students from Mexico estimated a prevalence of 5.2% [20], while in a multi-country study (in 10 countries including Peru) carried out by Király et al., the prevalence for the Peruvian population was 13.7% [47] (Table 2).

### 3.4. Results of Factors Associated with Gaming Disorder

Four of the six studies included evaluated factors associated with GD [20,21,22,23].

#### 3.4.1. Game-Related Factors

The study carried out by Ferreira et al. reported that predictors of GD were related to the number of video game genres played (first-person shooters, action games, role-playing games, social network games, puzzle/simulators, and other [board games and trivia]), number of non-stop hours of gaming, and proportion of time played online [21]. In addition, the study carried out by Severo et al. showed that people who spend more than half of their free time playing video games and with a certain weekly time spent playing games classified in hours (from 2 to 6 h, from 7 to 19 h, and more than 20 h compared to <1 h) were more likely to present GD [23] (Table 2).

#### 3.4.2. Sociodemographic and Health Risk Factors

Two studies (conducted by Chagas Brandão et al. and Severo et al.) reported that males were more likely to present GD [22,23]. On the other hand, the study carried out by Chagas Brandão et al. reported that smoking and alcohol consumption were factors associated with GD [22] (Table 2).

#### 3.4.3. Interpersonal Relationships and School and Work Performance

The study carried out by Borges et al. reported that people with serious health-related impairment in domains related to administration or housework, college and other jobs, close personal relationships, and social life were more likely to have GD [20]. In addition, the study carried out by Chagas Brandão et al. showed that people with relationship problems with their partners have a higher probability of suffering from GD [22] (Table 2).

#### 3.4.4. Personality, Psychiatric Comorbidity, and Physical Health Conditions

The study carried out by Borges et al. determined that people who have psychological, medical, or any type of treatment throughout life are more likely to present GD [20]. The study conducted by Chagas Brandão et al. reported that people who have behaviors of perpetration and victimization of bullying, hyperactivity/inattention, prosocial behavior, conduct problems, and the presence of emotional symptoms were more likely to present GD [22]. Likewise, the study carried out by Ferreira et al. reported that the presence of any mental disorder was a predictor of GD in people. On the other hand, the study carried out by Severo et al. reported that severe depression and poorer sleep quality were significantly associated with the presence of GD [21] (Table 2).

### 3.5. Quality Assessment of the Studies Included

In relation to the evaluation of the methodological quality of the studies included, it was found that the study of Chagas Brandão et al. had an affirmative response (Yes) in all the items that make up the JBI Critical Appraisal Checklist [22]. Likewise, the study conducted by Ferreira et al. had an affirmative response in most of the items except for item 4 [21], which refers to the way of measuring the outcome and the objective of the study, while the study by Severo et al. had only a negative response in item 2 [23], which refers to the detailed description of the participants and settings of the study. Finally, the studies by Andrade et al. [19] and Király et al. [47] had the highest number of negative responses (No, Not Applicable, or unclear) in the items that make up the JBI Critical Appraisal Checklist (Table 3).

## 4. Discussion

This review aimed to determine the prevalence and factors associated with GD in the LAC population. Regarding the documents identified in the databases, only six studies that measured the presence of this disorder in the LAC population were found (three studies in Brazil [21,22,23], one in Ecuador [19], México [20] and Peru [47]). All studies were cross-sectional. The prevalence of GD in LAC ranged from 1.1% to 38.2%, with the highest prevalence reported in studies conducted in Brazil. Concerning the factors associated with GD (four of the six studies identified evaluated these factors), the type of game, the pattern of use (hours played), being male, consuming tobacco and alcohol, having poor interpersonal relationships, and the presence of mental disorders were related to the presence of this disorder.

In relation to the prevalence of GD, a systematic review published with studies up to 2019 indicated that 3.05% of the population worldwide has GD, with variability in the prevalence due to the population sampled and the criteria used to define GD [4]. This review did not include studies in LAC countries. Another systematic review (which also included the study in the population of Mexico evaluated in our study), including studies up to 3 December 2020, reported a prevalence of GD of 3.03% [53]. The high variability of instruments to define GD used in the different studies available in the literature demonstrates that the values of the prevalence of GD, their interpretation, and applicability in decision making in health should be considered with caution [54]. Although the prevalence of GD in LAC populations described is higher than these values, interpretation should be made with caution for the reasons described. On the other hand, the lack of studies on GD in LAC identified in this review and global reviews on GD demonstrates the limited evidence available on this problem in the region and the need to develop further studies. A potential reason for why new studies on the prevalence of GD in LAC and other regions of the world have not been conducted could be attributed to the COVID-19 pandemic, which reoriented the development of research in the generation of knowledge about this disease, reducing and limiting the development of research in other health topics as well [55]. Several authors discussed the relevance and implications of the inclusion of GD within the ICD-11 diagnoses in the last two years [56,57]. The expected development of future research using a standard clinical criterion (ICD-11 will come into effect in 2022) for GD classification will allow more comparable measurements among studies as well as greater applicability of the results of the prevalence of this problem for the development of programs and strategies aimed at reducing this disorder in the LAC population.

Regarding the factors associated with GD in LAC, it should be noted that only four of the six studies evaluated the factors that influence GD in this region of the world [20,21,22,23]. In relation to the factors identified, characteristics regarding the game (type), pattern of use, as well as gender (higher in men), tobacco and alcohol consumption, poor interpersonal relationships, and the presence of mental disorders were found to be associated with GD in LAC. In this regard, previous studies in adolescents from Spain and Korea and in adults from the latter country also reported that mental health problems, especially anxiety, and time spent playing games were associated with an increase in the presence of GD [58,59]. The characteristics described in these last two countries were also reported in a meta-analysis on GD of studies in a Chinese population [60]. Another characteristic described as being related to GD is family dynamics and the type of interpersonal relationships experienced from an early age, with dysfunctional families and inadequate interpersonal relationships increasing the presence of GD [61]. Smoking and alcohol consumption are described as the main factors associated with chronic diseases in the LAC region [62,63], and the presence of dysfunctional families and poor personal relationships have also been described in this population. These factors generate imbalances in reward circuits and predisposition to addiction problems such as GD [64], making it necessary to identify subgroups of the population that may present a higher risk of GD to prioritize efforts to control this condition in settings with limited resources such as those of the LAC region.

A meta-analysis was not performed in this study on the prevalence of GD in LAC and its associated factors. As described in the Cochrane Handbook [65], due to differences in the measurement of the outcome as well as reviews in which the primary studies may present a high possibility of bias, it would not be appropriate to carry out a meta-analysis. Since the studies analyzed included different populations (schoolchildren, university students, and users of an online platform) and the use of different instruments for the identification of GD (IGD20, DSM-5, GAS, IGDT-10, IGDS9-SF, and Self-report instrument based on the DSM-5 IGD criteria), the authors did not consider it appropriate to perform a meta-analysis to pool the results of the studies included in this review. In the systematic review with the meta-analysis by Stevens et al. on the global prevalence of GD, the variability of the estimates on the prevalence of GD was described to be influenced by the instrument or criteria used to define a case of GD, with a reported prevalence of 77% in that study. Likewise, the use of some instruments for the measurement of GD led to higher prevalence values of this disorder [4]. Similarly, lower cut-off points in the scales to define GD, samples that include adolescents, as well as small samples have been related to a higher prevalence of GD [4]. All of the above could explain the great variability in the prevalence described in the studies that evaluated GD in LAC, with three of the six studies identified being carried out in adolescents [19,21,22], two others in a young population [20,23], one in adults [47], and six different instruments were applied to identify cases of GD (IGD20; DSM-5; GAS; IDT-10, IGDS9–SF and Self-report instrument based on the DSM-5 IGD criteria).

This review has some limitations. Although an exhaustive search of documents was carried out in different bibliographic databases to identify studies on GD and its associated factors in LAC, including a bibliographic database of research in the LAC region (LILACS) and one focused on studies of psychology (PsycINFO), there may be articles on GD in LAC available in the gray literature or other regional repositories that were not identifiable with the methodology used. Additionally, the GD prevalence reported in the studies included is based on self-report data, which could not reflect GD statistics based on clinical diagnosis. In addition, there was variability regarding the GD measurement instrument, the analyses performed to identify factors associated with GD, as well as the way of presenting the results. Likewise, in general, the studies evaluated did not require information on the sociodemographic characteristics of the participants, specifically on the socioeconomic level or urban or rural area of residence, which are characteristics that can influence the access and use of the Internet and technologies, and thus, the prevalence of GD. Additionally, some studies were included despite not having a perfect quality assessment. Therefore, some studies may not report fully reliable data. Taking the above into account, it was not considered appropriate to carry out a meta-analysis, as proposed in the protocol registered in PROSPERO for this systematic review. Despite the limitations, this systematic review was compiled by synthesizing the evidence available on GD and its associated factors in LAC. We performed exhaustive searches to identify evidence on the subject in bibliographic databases widely used in this type of review, without the restriction of language, thereby contributing to knowledge since, to the knowledge of the authors, this is the first systematic review on this disorder in the region.

This systematic review also makes some contributions to the literature. These findings could indicate that GD is a prevalent disorder in the LAC region. However, as it is a recent pathology (because the year of inclusion of GD was 2013), the health systems of the countries involved must develop new public health policies based on the factors associated with improving the quality of life of these patients. Although further evidence is needed, the present study contributes to a better understanding of this diagnosis, its prevalence, and the factors associated in a region of middle-to-high income countries, such as LAC.

In conclusion, this review found limited evidence on the prevalence of GD and its associated factors in the LAC population. Factors related to the type and hours of game use, male gender, tobacco and alcohol consumption, poor interpersonal relationships, and the presence of mental disorders were found to increase the probability of presenting GD. GD can lead to problems in the quality of life of the person with this condition. Therefore, it is necessary to develop strategies for the detection of this disorder focusing on population subgroups at higher risk to identify cases as well as develop effective interventions to control this problem. Within the context of the COVID-19 pandemic, an increase in the use and time spent on games and a consequent increase in this problem is expected, especially in the young population. Therefore, it is necessary to identify patients who developed GD during the pandemic, and to study the effect of health control measures, including isolation, on the development of GD and related problems.

## Figures and Tables

**Table 1:** Characteristics of the studies included.

Author (Year)	Journal	Country	Study Design	Data Collection Period	Data Collection Method	Sampling Method	Setting	Number of Settings	Sample Characteristics	Gamer Characteristics	Male (%)	Age (Years) (Mean [SD] or Range)
Andrade et al. (2018) [19]	Health and Addictions	Ecuador	Cross-sectional	NR	Face-to-face and online questionnaire	Non-Probability	Educational institutions	76	School students	NR	52.7	15.62 (±0.8)
Borges et al. (2019) [20]	Journal of Behavioral Addictions	Mexico	Cross-sectional	January 2018 to February 2019	Online questionnaire	Non-Probability	University	5	University students	NR	44.6	18–19 (NR) and 20 or more (NR)
Chagas Brandão et al. (2021) [22]	Journal of Addictive Diseases	Brazil	Cross-sectional	February–March, 2019 to August–September 2020	Face-to-face questionnaire	Probability	Public schools	70	School students	NR	49.4	13.2 (±0.8)
Ferreira et al. (2021) [21]	Brazilian Journal of Psychiatry	Brazil	Cross-sectional	NR	Face-to-face questionnaire	Probability	Schools in general	57	School students	NR	60.9	14.3 (SD: 1.9)
Király et al. (2019) [47]	Psychology of Addictive Behaviors	Peru	Cross-sectional	April to July, 2015	Online questionnaire	NR	Online platform	NR	Gamer sample	NR	98.7	21.3 (SD: 3.3)
Severo et al. (2020) [23]	Brazilian Journal of Psychiatry	Brazil	Cross-sectional	October and November, 2017	Face-to-face questionnaire	NR	Schools and universities	NR	School and university students	Past-year gamers	57.5	20.3 (SD: 5.4)

SD: standard deviation, NR: not reported.

**Table 2:** Prevalence and factors associated with gaming disorder in Latin America and the Caribbean.

Author (Year)	Instrument Used	Cut-Off for GD	GD Cases	Sample Size (*n*)	Prevalence of GD (%)	Factors Associated with GD
Andrade et al. (2018) [19]	IGD20	≥75 points	36	3178	1.13	NR
Borges et al. (2019) [20]	DSM-5 IGD scale	Presence of five out of nine symptoms	367	7022	5.2	Lifetime psychological treatment: aOR: 1.9; 95% CI: 1.4–2.4
						Lifetime medical treatment: aOR: 1.8; 95% CI: 1.1–3.0
						Lifetime any treatment: aOR: 1.8, 95% CI: 1.4–2.4
						Severe impairment–home: aOR: 2.1; 95% CI: 1.1–3.8
						Severe impairment–work/school: aOR: 2.6; 95% CI: 1.7–4.1
						Severe impairment–relationships: aOR: 1.8; 95% CI: 1.1–2.8
						Severe impairment–social: aOR: 1.9, 95% CI: 1.3–3.0
						Severe impairment–tota: aOR: 2.4, 95% CI: 1.7–3.3
Chagas Brandão et al. (2021) [22]	Self-report instrument based on the DSM-5 IGD criteria	Presence of five out of nine symptoms	1077	3939	28.2	Male: aOR: 3.43; 95% CI: 3.03–3.89
						Tobacco use: aOR: 1.20; 95% CI: 1.01–0.44
						Alcohol use: aOR: 1.29; 95% CI: 1.16–1.43
						Bullying Perpetration: aOR: 1.29; 95% CI: 1.16–1.43
						Bullying Victimization: aOR: 1.29; 95% CI: 1.16–1.43
						Hyperactivity/Inattention: aOR: 1.29; 95% CI: 1.16–1.43
						Prosocial Behavior: aOR: 1.29; 95% CI: 1.16–1.43
						Conduct Problems: aOR: 1.29; 95% CI: 1.16–1.43
						Peer Relationship Problems: aOR: 1.29; 95% CI: 1.16–1.43
						Emotional Symptoms: aOR: 1.29; 95% CI: 1.16–1.43
Ferreira et al. (2021) [21]	GAS	3 or more on at least four questions	83	407	20.4	Number of genres played: Est. = 0.43, *p* < 0.001
						Number of non-stop hours: Est. = 0.2, *p* < 0.05
						Proportion of time played online: Est. = 0.31, *p* < 0.05
						Presence of any mental disorder: Est. = 1.4, *p* < 0.001
Király et al. (2019) [47]	IGDT-10	5 or more points	NR	612	13.7	NR
Severo et al. (2020) [23]	IGDS9-SF	Moderate risk for GD: >16 points; High risk for GD: >21 points	Moderate or high: 212 (high: 101)	555	Moderate or high: 38.2 (high: 18.2)	Gender: OR: 2.18; 95% CI: 1.34–3.60
						Sleep Pittsburgh Sleep Quality Index: OR: 1.78; 95% CI: 1.08–2.93
						Severe Depression: OR: 16.30; CI: 3.61–73.59
						More than half of the free time spent on video games: OR: 2.88; 95% CI: 1.73–4.80
						Weekly time spent gaming 2–6 h: OR: 4.89; 95% CI: 2.49–9.61
						Weekly time spent gaming 17–19 h: OR: 7.83, 95% CI: 3.65–16.81
						Weekly time spent gaming >20 h: OR: 13.47; 95% CI: 5.64–32.19

GD: gaming disorder, OR: odds ratio, aOR: adjusted odds ratio, CI: confidence interval; IGD20: Internet Gaming Disorder Test, DSM-5: Diagnostic and Statistical Manual of Mental Disorders, 5th Edition, GAS: Gaming Addiction Scale, IGDT-10: Ten-Item Internet Gaming Disorder Test, IGDS9-SF: Brazilian version of the Internet Gaming Disorder Scale-Short-Form, Est: estimate, NR: not reported. GD prevalence reported in the studies being included is based on self-report data, which could not reflect GD statistics based on clinical diagnosis.

**Table 3:** Evaluation of the quality of the studies included.

Author (Year)	Q1	Q2	Q3	Q4	Q5	Q6	Q7	Q8
Andrade et al. (2018) [19]	No	Yes	Yes	Unclear	No	No	Yes	No
Borges et al. (2019) [20]	No	No	Yes	Unclear	Yes	Yes	Yes	Yes
Chagas Brandão et al. (2021) [22]	Yes	Yes	Yes	Yes	Yes	Yes	Yes	Yes
Ferreira et al. (2021) [21]	Yes	Yes	Yes	Unclear	Yes	Yes	Yes	Yes
Király et al. (2019) [47]	Unclear	Unclear	NA	Yes	NA	NA	Yes	Yes
Severo et al. (2020) [23]	Yes	Unclear	Yes	Yes	Yes	Yes	Yes	Yes

NA: Not Applicable. Q1: Were the criteria for inclusion in the sample clearly defined? Q2: Were the study subjects and the setting described in detail? Q3: Was the exposure measured in a valid and reliable way? Q4: Were objective, standard criteria used for measurement of the condition? Q5: Were confounding factors identified? Q6: Were strategies to deal with confounding factors stated? 7: Were the outcomes measured in a valid and reliable way? Q8: Was appropriate statistical analysis used?

## Supplementary Materials

The following supporting information can be downloaded at: https://www.mdpi.com/article/10.3390/ijerph191610036/s1. Table S1: Search Strategies.
