# Supplementary material for: Prevalence and Factors Associated with Gaming Disorder in Latin America and the Caribbean: A Systematic Review
Source: Int J Environ Res Public Health. 2022 Aug 15;19(16):10036. doi: 10.3390/ijerph191610036 (PMC9408645; doi:10.3390/ijerph191610036)
Supplement: Supplementary file 1 [file ijerph-19-10036-s001.zip › ijerph-1746597-supplementary.pdf]

Supplementary Material

Supplementary Table S1. Search Strategies.

**Database**	**PubMed**<br>**Date:** 29/12/2021		**Results**
**Search Strategy**	#1	Video Games[Majr] Sort by: First Author	4,977
	#2	Computer Gam*[tiab] Sort by: First Author	1,486
	#3	Video Gam*[tiab] Sort by: First Author	4,35
	#4	Online Gam*[tiab] Sort by: First Author	903
	#5	Internet Gam*[tiab] Sort by: First Author	1,114
	#6	Gamer*[tiab] Sort by: First Author	869
	#7	Virtual Gam*[tiab] Sort by: First Author	95
	#8	Xbox[tiab] Sort by: First Author	1,211
	#9	PlayStation*[tiab] Sort by: First Author	85
	#10	Play Station[tiab] Sort by: First Author	1
	#11	Nintendo[tiab] Sort by: First Author	602
	#12	Multiplayer Online[tiab] Sort by: First Author	134
	#13	Battle Arena*[tiab] Sort by: First Author	18
	#14	MOBA[tiab] Sort by: First Author	539
	#15	MMORPG[tiab] Sort by: First Author	45
	#16	#1 OR #2 OR #3 OR #4 OR #5 OR #6 OR #7 OR #8 OR #9 OR #10 OR #11 OR #12 OR #13 OR #14 OR #15 Sort by: First Author	11,664
	#17	Technology Addiction[Mesh] Sort by: First Author	417
	#18	Abuse*[tiab] Sort by: First Author	146,323
	#19	Addict*[tiab] Sort by: First Author	73,35
	#20	Compulsive*[tiab] Sort by: First Author	25,135
	#21	Dependenc*[tiab] Sort by: First Author	298,109
	#22	Disorder*[tiab] Sort by: First Author	1,296,204
	#23	Effect*[tiab] Sort by: First Author	7,652,609
	#24	Excessive*[tiab] Sort by: First Author	149,415
	#25	Habit*[tiab] Sort by: First Author	199,65
	#26	Misuse*[tiab] Sort by: First Author	23,367
	#27	Pathologic*[tiab] Sort by: First Author	509,394
	#28	Problem*[tiab] Sort by: First Author	1,138,466
	#29	#17 OR #18 OR #19 OR #20 OR #21 OR #22 OR #23 OR #24 OR #25 OR #26 OR #27 OR #28 Sort by: First Author	10,136,673
	#30	(Americas[MeSH Terms:noexp] OR Latin America[Mesh] OR Latin America*[tiab] OR Latinamerica*[tiab] OR Latinoamerica*[tiab] OR Hispanoamerica[tiab] OR Iberoamerica*[tiab] OR Ibero Americ*[tiab] OR Panamerican*[tiab] OR Central America[Mesh] OR Central America*[tiab] OR Centroamerica*[tiab] OR	1,259,442

		Mesoamerica*[tiab] OR Meso America*[tiab] OR Middle America*[tiab] OR South America[Mesh] OR South America*[tiab] OR Southamerica*[tiab] OR Sudamerica*[tiab] OR "America del sur"[tiab] OR Caribbean Region[Mesh] OR Caribbean[tiab] OR Caribe*[tiab] OR West Indies[Mesh] OR West Indi*[tiab] OR Antill*[tiab] OR Indians, South American[Mesh] OR Indians, Central American[Mesh] OR Amerindian*[tiab] OR Indians[tiab] OR American Indian*[tiab] OR Native America*[tiab] OR Patagoni*[tiab] OR Andes[tiab] OR Andean*[tiab] OR Amazon*[tiab] OR Argentin*[ad] OR Argentin*[tiab] OR Argentina[pl] OR Bolivia*[ad] OR Bolivia*[tiab] OR Bolivia[pl] OR Brazil*[ad] OR Brasil*[ad] OR Brazil*[tiab] OR Brasil*[tiab] OR Brazil[pl] OR Colombia*[ad] OR Colombia*[tiab] OR Colombia[pl] OR Chile*[ad] OR Chile*[tiab] OR Chile[pl] OR Ecuador*[ad] OR Ecuator*[ad] OR Ecuador*[tiab] OR Ecuador[pl] OR Guiana*[ad] OR Guiana*[tiab] OR French Guiana[pl] OR Guyan*[ad] OR Guyan*[tiab] OR Guyana[pl] OR Paraguay*[ad] OR Paraguay*[tiab] OR Paraguay[pl] OR Peru*[ad] OR Peru*[tiab] OR Peru[pl] OR Surinam*[ad] OR Surinam*[tiab] OR Surinam*[pl] OR Uruguay*[ad] OR Uruguay*[tiab] OR Uruguay[pl] OR Venez*[ad] OR Venez*[tiab] OR Venezuela[pl] OR Belize*[ad] OR Belize*[tiab] OR Belize[pl] OR Costa Ric*[ad] OR Costarric*[ad] OR Costaric*[ad] OR Costa Ric*[tiab] OR Costarric*[tiab] OR Costaric*[tiab] OR Costa Rica[pl] OR Salvador*[ad] OR Salvador*[tiab] OR El Salvador[pl] OR Guatemal*[ad] OR Guatemal*[tiab] OR Guatemala[pl] OR Hondur*[ad] OR Hondur*[tiab] OR Honduras[pl] OR Nicaragu*[ad] OR Nicaragu*[tiab] OR Nicaragua[pl] OR Panam*[ad] OR Panam*[tiab] OR Panama[pl] OR Mexico[Mesh] OR Mexic*[ad] OR Mexic*[tiab] OR Mejic*[tiab] OR Mexico[pl] OR Baham*[ad] OR Baham*[tiab] OR Bahamas[pl] OR Cuba*[ad] OR Cuba*[tiab] OR Cuba[pl] OR Dominic*[ad] OR Dominic*[tiab] OR Dominican Republic[pl] OR Haiti*[ad] OR Haiti*[tiab] OR Haiti[pl] OR Jamaic*[ad] OR Jamaic*[tiab] OR Jamaica[pl] OR Puerto Rico[Mesh] OR Puerto Ric*[tiab] OR Puertorric*[tiab] OR Puertoric*[tiab]) Sort by: First Author	
	#31	#16 AND #29 AND #30 Sort by: First Author	322

Database	EMBase (Ovid)<br>**Date:** 30/12/2021		Results
**Search Strategy**	#1	exp video game/	4667
	#2	(Computer adj3 Gam*).ti,ab.	2921
	#3	(Video adj3 Gam*).ti,ab.	5365
	#4	(Online adj3 Gam*).ti,ab.	1543
	#5	(Internet adj3 Gam*).ti,ab.	1858
	#6	(Virtual adj3 Gam*).ti,ab.	946
	#7	Gamer*.ti,ab.	847
	#8	Xbox.ti,ab.	320
	#9	PlayStation*.ti,ab.	117
	#10	(Play adj1 Station).ti,ab.	18
	#11	Nintendo.ti,ab.	886
	#12	(Multiplayer adj3 Online).ti,ab.	177
	#13	Battle Arena*.ti,ab.	17
	#14	MOBA.ti,ab.	622
	#15	MMORPG*.ti,ab.	80
	#16	or/1-15	14416
	#17	exp game addiction/	1455
	#18	Abuse*.ti,ab.	189238
	#19	Addict*.ti,ab.	101589
	#20	Compulsive*.ti,ab.	34148
	#21	Dependenc*.ti,ab.	337052
	#22	Disorder*.ti,ab.	1741153
	#23	Effect*.ti,ab.	9798022
	#24	Excessive*.ti,ab.	216675
	#25	Habit*.ti,ab.	244682
	#26	Misuse*.ti,ab.	30633
	#27	Pathologic*.ti,ab.	769075
	#28	Problem*.ti,ab.	1500512
	#29	or/17-28	13074714
	#30	(exp South/ and Central America/) or (Latin adj1 America*).ti,ab. or Latinamerica*.ti,ab. or Latinoamerica*.ti,ab. or Hispanoamerica.ti,ab. or Iberoamerica*.ti,ab. or (Ibero adj1 Americ*).ti,ab. or Panamerica*.ti,ab. or (South adj1 America*).ti,ab. or Southamerica*.ti,ab. or Sudamerica*.ti,ab. or (America adj1 Sur).ti,ab. or (Central adj1 America*).ti,ab. or Centroamerica*.ti,ab. or Mesoamerica*.ti,ab. or (Meso adj1 America*).ti,ab. or (Middle adj1 America*).ti,ab. or exp Caribbean Islands/ or Caribbean*.ti,ab. or Caribe*.ti,ab. or (West adj1 Indi*).ti,ab. or Antill*.ti,ab. or exp American indian/ or Amerindian*.ti,ab. or Indians.ti,ab. or (Native adj1 America*).ti,ab. or Patagoni*.ti,ab. or Andes.ti,ab. or Andean*.ti,ab. or Amazon*.ti,ab. or exp Argentina/ or Argentin*.ti,ab. or exp Bolivia/ or Bolivia*.ti,ab. or exp Brazil/ or Brazil*.ti,ab. or Brasil*.ti,ab. or exp Colombia/ or Colombia*.ti,ab. or exp Chile/ or Chile*.ti,ab. or exp Ecuador/ or Ecuador*.ti,ab. or exp French Guiana/ or Guiana*.ti,ab. or exp Guyana/ or Guyan*.ti,ab. or exp Paraguay/ or Paraguay*.ti,ab. or exp Peru/ or Peru*.ti,ab. or exp Suriname/ or Surinam*.ti,ab. or exp Uruguay/ or Uruguay*.ti,ab. or exp Venezuela/ or Venez*.ti,ab. or exp Belize/ or Beliz*.ti,ab. or exp Costa Rica/ or (Costa adj1 Rica).ti,ab. or	570109

		Costarric*.ti,ab. or Costaric*.ti,ab. or exp El salvador/ or Salvador*.ti,ab. or exp Guatemala/ or Guatemal*.ti,ab. or exp Honduras/ or Hondur*.ti,ab. or exp Nicaragua/ or Nicaragu*.ti,ab. or exp Panama/ or Panam*.ti,ab. or exp Mexico/ or Mexic*.ti,ab. or exp Cuba/ or Cuba*.ti,ab. or exp Dominican Republic/ or Dominica*.ti,ab. or exp Haiti/ or Haiti*.ti,ab. or exp Jamaic/ or Jamaic*.ti,ab. or exp Puerto Rico/ or (Puerto adj1 Ric*).ti,ab. or Puertoric*.ti,ab. or Puertorric*.ti,ab.	
	#31	16 and 29 and 30	152

Database	Global Health (OVID)<br>**Date:** 30/12/2021		**Results**
**Search Strategy**	#1	video game/	247
	#2	(Computer adj3 Gam*).ti,ab.	384
	#3	(Video adj3 Gam*).ti,ab.	717
	#4	(Online adj3 Gam*).ti,ab.	149
	#5	(Internet adj3 Gam*).ti,ab.	198
	#6	(Virtual adj3 Gam*).ti,ab.	39
	#7	Gamer*.ti,ab.	85
	#8	Xbox.ti,ab.	21
	#9	PlayStation*.ti,ab.	8
	#10	(Play adj1 Station).ti,ab.	1
	#11	Nintendo.ti,ab.	44
	#12	(Multiplayer adj3 Online).ti,ab.	9
	#13	Battle Arena*.ti,ab.	2
	#14	MOBA.ti,ab.	227
	#15	MMORPG*.ti,ab.	4
	#16	or/1-15	1568
	#17	Abuse*.ti,ab.	21494
	#18	Addict*.ti,ab.	10269
	#19	Compulsive*.ti,ab.	1483
	#20	Dependenc*.ti,ab.	23253
	#21	Disorder*.ti,ab.	140630
	#22	Effect*.ti,ab.	1424740
	#23	Excessive*.ti,ab.	30713
	#24	Habit*.ti,ab.	77109
	#25	Misuse*.ti,ab.	5106
	#26	Pathologic*.ti,ab.	50518
	#27	Problem*.ti,ab.	218441
	#28	or/17-27	1771317
	#29	(Latin America* or Latinamerica* or Latinoamerica* or Latin* or Hispanic Americans or Iberoamerica* or Ibero Americ* or Panamerican* or Central America* or Centroamerica* or Mesoamerica* or Meso America* or Middle America* or South America* or Southamerica* or Sudamerica* or America del sur or Caribbean or Caribe* or West Indi* or Antill* or Amerindian* or Indians or American Indian* or Native America* or Patagoni* or Andes or Andean* or Amazon* or Argentin* or Bolivia* or Brazil* or Brasil* Colombia* or Colombia* or Colombia or Chile* or Ecuador* or Guiana* or Guyan* or Guyan* or Paraguay* or Paraguay* or Peru* or Surinam* or Surinam* or Uruguay* or Venez* or Belize* or Costa Ric* or Costarric* or Costaric* or Costa Ric* or Costarric* OR Salvador* or Salvador* or Salvador or Guatemal* or Guatemal* or Guatemal a or Hondur* or Nicaragu* Panam* or Mexic* or Cuba* or Dominic* or Dominic* or Haiti* or Jamaic* or Puerto Ric* or Puertorric* or Puertoric*).ti,ab.	23528

	#30	16 and 28 and 29	47

Database	PsycINFO (OVID)<br>**Date:** 30/12/2021		Results
**Search Strategy**	#1	exp Computer Games/	8341
	#2	(Computer adj3 Gam*).ti,ab.	2814
	#3	(Video adj3 Gam*).ti,ab.	5971
	#4	(Online adj3 Gam*).ti,ab.	2500
	#5	(Internet adj3 Gam*).ti,ab.	1550
	#6	(Virtual adj3 Gam*).ti,ab.	761
	#7	Gamer*.ti,ab.	1320
	#8	Xbox.ti,ab.	103
	#9	PlayStation*.ti,ab.	57
	#10	(Play adj1 Station).ti,ab.	6
	#11	Nintendo.ti,ab.	278
	#12	(Multiplayer adj3 Online).ti,ab.	552
	#13	Battle Arena*.ti,ab.	30
	#14	MOBA.ti,ab.	98
	#15	MMORPG*.ti,ab.	258
	#16	or/1-15	14679
	#17	exp internet addiction/	3100
	#18	Abuse*.ti,ab.	130324
	#19	Addict*.ti,ab.	56001
	#20	Compulsive*.ti,ab.	28558
	#21	Dependenc*.ti,ab.	75552
	#22	Disorder*.ti,ab.	532173
	#23	Effect*.ti,ab.	1461060
	#24	Excessive*.ti,ab.	26822
	#25	Habit*.ti,ab.	57979
	#26	Misuse*.ti,ab.	13936
	#27	Pathologic*.ti,ab.	43652
	#28	Problem*.ti,ab.	559826
	#29	or/17-28	2351856
	#30	(exp South/ and Central America/) or (Latin adj1 America*).ti,ab. or Latinamerica*.ti,ab. or Latinoamerica*.ti,ab. or Hispanoamerica.ti,ab. or Iberoamerica*.ti,ab. or (Ibero adj1 Americ*).ti,ab. or Panamerica*.ti,ab. or (South adj1 America*).ti,ab. or Southamerica*.ti,ab. or Sudamerica*.ti,ab. or (America adj1 Sur).ti,ab. or (Central adj1 America*).ti,ab. or Centroamerica*.ti,ab. or Mesoamerica*.ti,ab. or (Meso adj1 America*).ti,ab. or (Middle adj1 America*).ti,ab. or exp Caribbean Islands/ or Caribbean*.ti,ab. or Caribe*.ti,ab. or (West adj1 Indi*).ti,ab. or Antill*.ti,ab. or exp American indian/ or Amerindian*.ti,ab. or Indians.ti,ab. or (Native adj1 America*).ti,ab. or Patagoni*.ti,ab. or Andes.ti,ab. or Andean*.ti,ab. or Amazon*.ti,ab. or exp Argentina/ or Argentin*.ti,ab. or exp Bolivia/ or Bolivia*.ti,ab. or exp Brazil/ or Brazil*.ti,ab. or Brasil*.ti,ab. or exp Colombia/ or Colombia*.ti,ab. or exp Chile/ or Chile*.ti,ab. or exp Ecuador/ or Ecuador*.ti,ab. or exp French Guiana/ or Guiana*.ti,ab. or exp Guyana/ or Guyan*.ti,ab. or exp Paraguay/ or Paraguay*.ti,ab. or exp Peru/ or Peru*.ti,ab. or exp Suriname/ or Surinam*.ti,ab. or exp Uruguay/ or Uruguay*.ti,ab. or exp Venezuela/ or Venez*.ti,ab. or exp Belize/ or Beliz*.ti,ab. or exp Costa Rica/ or (Costa adj1 Rica).ti,ab. or	112230

		Costarric*.ti,ab. or Costaric*.ti,ab. or exp El salvador/ or Salvador*.ti,ab. or exp Guatemala/ or Guatemal*.ti,ab. or exp Honduras/ or Hondur*.ti,ab. or exp Nicaragua/ or Nicaragu*.ti,ab. or exp Panama/ or Panam*.ti,ab. or exp Mexico/ or Mexic*.ti,ab. or exp Cuba/ or Cuba*.ti,ab. or exp Dominican Republic/ or Dominica*.ti,ab. or exp Haiti/ or Haiti*.ti,ab. or exp Jamaic/ or Jamaic*.ti,ab. or exp Puerto Rico/ or (Puerto adj1 Ric*).ti,ab. or Puertoric*.ti,ab. or Puertorric*.ti,ab.	
	#31	16 and 29 and 30	102

**Database**	**CINAHL (Complete-EBSCO)**<br>**Date:** 30/12/2021		**Results**
**Search Strategy**	#1	(MH "Video Games+")	5,374
	#2	TI (Computer N3 Gam*) OR AB (Computer N3 Gam*)	1,038
	#3	TI (Video N3 Gam*) OR AB (Video N3 Gam*)	2,746
	#4	TI (Online N3 Gam*) OR AB (Online N3 Gam*)	830
	#5	TI (Internet N3 Gam*) OR AB (Internet N3 Gam*)	708
	#6	TI (Virtual N3 Gam*) OR AB (Virtual N3 Gam*)	491
	#7	TI Gamer* OR AB Gamer*	401
	#8	TI Xbox OR AB Xbox	142
	#9	TI PlayStation* OR AB PlayStation*	49
	#10	TI (Play N1 Station*) OR AB (Play N1 Station*)	10
	#11	TI Nintendo OR AB Nintendo	454
	#12	TI (Multiplayer N3 Online) OR AB (Multiplayer N3 Online)	106
	#13	TI (Battle N1 Arena*) OR AB (Battle N1 Arena*)	14
	#14	TI MOBA OR AB MOBA	167
	#15	TI MMORPG* OR AB MMORPG*	48
	#16	S1 OR S2 OR S3 OR S4 OR S5 OR S6 OR S7 OR S8 OR S9 OR S10 OR S11 OR S12 OR S13 OR S14 OR S15	8,213
	#17	(MH "Internet Addiction+")	1,156
	#18	TI Abuse* OR AB Abuse*	63,269
	#19	TI Addict* OR AB Addict*	28,021
	#20	TI Compulsive* OR AB Compulsive*	6,999
	#21	TI Dependenc* OR AB Dependenc*	37,421
	#22	TI Disorder* OR AB Disorder*	308,88
	#23	TI Effect* OR AB Effect*	1,273,146
	#24	TI Excessive* OR AB Excessive*	27,821
	#25	TI Habit* OR AB Habit*	38,917
	#26	TI Misuse* OR AB Misuse*	11,228
	#27	TI Pathologic* OR AB Pathologic*	61,063
	#28	TI Problem* OR AB Problem*	296,665
	#29	S17 OR S18 OR S19 OR S20 OR S21 OR S22 OR S23 OR S24 OR S25 OR S26 OR S27 OR S28	1,833,755
	#30	TI (Latin NEAR/1 America*) OR Latinamerica* OR Latinoamerica* OR Latin* OR Hispanic Americans OR Iberoamerica* OR (Ibero NEAR/1 Americ*) OR Panamerican* OR (Central NEAR/1 America*) OR Centroamerica* OR Mesoamerica* OR (Meso NEAR/1 America*) OR (Middle NEAR/1 America*) OR (South NEAR/1 America*) OR Southamerica* OR Sudamerica* OR (America NEAR/1 Sur)	62,646

		OR Caribbean OR Caribe* OR (West NEAR/1 Indi*) OR Antill* OR Amerindian* OR Indians OR (American NEAR/1 Indian*) OR (Native NEAR/1 America*) OR Patagoni* OR Andes OR Andean* OR Amazon* OR Argentin* OR Bolivia* OR Brazil* OR Brasil* Colombia* OR Colombia* OR Colombia OR Chile* OR Ecuador* OR Guiana* OR Guyan* OR Guyan* OR Paraguay* OR Paraguay* OR Peru* OR Surinam* OR Surinam* OR Uruguay* OR Venez* OR Belize* OR (Costa NEAR/1 Ric*) OR Costarric* OR Costaric* OR Costa Ric* OR Costarric* OR Salvador* OR Salvador* OR Guatemal* OR Guatemal* OR Guatemala OR Hondur* OR Nicaragu* OR Panam* OR Mexic* OR Cuba* OR Dominic* OR Dominic* OR Haiti* OR Jamaic* OR (Puerto NEAR/1 Ric*) OR Puertorric* OR Puertoric*	
	#31	AB (Latin NEAR/1 America*) OR Latinamerica* OR Latinoamerica* OR Latin* OR Hispanic Americans OR Iberoamerica* OR (Ibero NEAR/1 Americ*) OR Panamerican* OR (Central NEAR/1 America*) OR Centroamerica* OR Mesoamerica* OR (Meso NEAR/1 America*) OR (Middle NEAR/1 America*) OR (South NEAR/1 America*) OR Southamerica* OR Sudamerica* OR (America NEAR/1 Sur) OR Caribbean OR Caribe* OR (West NEAR/1 Indi*) OR Antill* OR Amerindian* OR Indians OR (American NEAR/1 Indian*) OR (Native NEAR/1 America*) OR Patagoni* OR Andes OR Andean* OR Amazon* OR Argentin* OR Bolivia* OR Brazil* OR Brasil* Colombia* OR Colombia* OR Colombia OR Chile* OR Ecuador* OR Guiana* OR Guyan* OR Guyan* OR Paraguay* OR Paraguay* OR Peru* OR Surinam* OR Surinam* OR Uruguay* OR Venez* OR Belize* OR (Costa NEAR/1 Ric*) OR Costarric* OR Costaric* OR Costa Ric* OR Costarric* OR Salvador* OR Salvador* OR Guatemal* OR Guatemal* OR Guatemala OR Hondur* OR Nicaragu* OR Panam* OR Mexic* OR Cuba* OR Dominic* OR Dominic* OR Haiti* OR Jamaic* OR (Puerto NEAR/1 Ric*) OR Puertorric* OR Puertoric*	95,013
	#32	S30 OR S31	119,755
	#33	S16 AND S29 AND S32	73

Database	Scopus<br>Date: 30/12/2021		Results
Search Strategy	#1	TITLE-ABS-KEY ("Video Games")	24,228
	#2	TITLE-ABS-KEY ("Computer Gam*")	18,393
	#3	TITLE-ABS-KEY ("Video Gam*")	24,739
	#4	TITLE-ABS-KEY ("Online Gam*")	6,91
	#5	TITLE-ABS-KEY ("Internet Gam*")	1,835
	#6	TITLE-ABS-KEY (Gamer*)	3,793
	#7	TITLE-ABS-KEY ("Virtual Gam*")	769
	#8	TITLE-ABS-KEY (Xbox)	993
	#9	TITLE-ABS-KEY ("PlayStation*")	632
	#10	TITLE-ABS-KEY ("Play Station")	74
	#11	TITLE-ABS-KEY ("Nintendo")	1,614
	#12	TITLE-ABS-KEY ("Multiplayer Online")	2,273
	#13	TITLE-ABS-KEY ("Battle Arena*")	198
	#14	TITLE-ABS-KEY ("MOBA")	917
	#15	TITLE-ABS-KEY ("MMORPG")	779
	#16	#1 OR #2 OR #3 OR #4 OR #5 OR #6 OR #7 OR #8 OR #9 OR #10 OR #11 OR #12 OR #13 OR #14 OR #15	52,765
	#17	TITLE-ABS-KEY ("Technology Addiction")	115
	#18	TITLE-ABS-KEY (Abuse*)	346,003
	#19	TITLE-ABS-KEY (Addict*)	175,176
	#20	TITLE-ABS-KEY (Compulsive*)	47,609
	#21	TITLE-ABS-KEY (Dependenc*)	1,542,150
	#22	TITLE-ABS-KEY (Disorder*)	3,123,970
	#23	TITLE-ABS-KEY (Effect*)	20,271,794
	#24	TITLE-ABS-KEY (Excessive*)	311,29
	#25	TITLE-ABS-KEY (Habit*)	652,947
	#26	TITLE-ABS-KEY (Misuse*)	53,063
	#27	TITLE-ABS-KEY (Pathologic*)	737,241
	#28	TITLE-ABS-KEY (Problem*)	6,032,657
	#29	#17 OR #18 OR #19 OR #20 OR #21 OR #22 OR #23 OR #24 OR #25 OR #26 OR #27 OR #28	28,765,135
	#30	TITLE-ABS-KEY ("latin america" OR "Latinoamerica" OR latin* OR "central america" OR "Centroamerica" OR "south America" OR sudamerica OR caribbean OR caribe* OR "west indies" OR antill* OR patagoni* OR andes OR andean OR amazon OR "Puerto rico" OR puertoric* OR puertorric* OR jamaica OR jamaic* OR haiti OR haiti* OR "dominican republic" OR dominica* OR cuba OR cuba* OR mexico OR mexic* OR mejic* OR panama OR panam* OR nicaragua OR nicaragu* OR honduras OR hondur* OR guatemala OR guatemal* OR "el Salvador" OR salvador* OR "costa rica" OR costarric* OR costaric* OR belize OR beliz* OR venezuela OR venez* OR uruguay OR uruguay* OR suriname OR surinam* OR peru OR peru* OR paraguay OR paraguay* OR guyana OR guyan* OR "french guiana" OR guiana* OR guayan* OR ecuador OR ecuador* OR chile OR chile* OR colombia OR colombia* OR brazil OR brazil* OR brasil* OR bolivia OR bolivia* OR argentina OR argentin*)	1,410,320

	#31	#16 AND #29 AND #30	378

**Database**	**LILACS (BVS – Eng)**<br>**Date:** 30/12/2021		**Results**
**Search Strategy**	#1	(MH Video Games OR ((Computer OR Computador$ OR Video OR Online OR Internet OR Virtual) AND (Game$ OR Gaming OR Juego$ OR Jogo$ OR Multiplayer$ OR Multijugador OR Multijogador)) OR Gamer$ OR Xbox OR PlayStation$ OR Nintendo OR ((Battle) AND (Arena$)) OR MOBA OR MMORPG$) AND (MH Technology Addiction OR Abuse$ OR Abuso$ OR Addict$ OR Adicción OR Adicto$ OR Vicio$ OR Compulsiv$ OR Dependenc$ OR Disorder$ OR Desorden$ OR Trastorno$ OR Effect$ OR Efecto$ OR Efeito$ OR Excessive$ OR Exces$ OR Habit$ OR Misuse$ OR Indebido$ OR Indevido$ OR Pathologic$ OR Patologic$ OR Problem$) [Words]	493
